# An activating mutation in the *CRHR1* gene is rarely associated with pituitary-dependent hyperadrenocorticism in poodles

**DOI:** 10.6061/clinics/2017(09)09

**Published:** 2017-09

**Authors:** Viviani De-Marco, Luciani R. Carvalho, Mariana F. Guzzo, Paulo S.L. Oliveira, Larissa G. Gomes, Berenice B. Mendonca

**Affiliations:** I Unidade de Endocrinologia do Desenvolvimento, Laboratorio de Hormonios e Genetica Molecular LIM/42, Faculdade de Medicina FMUSP, Universidade de Sao Paulo, Sao Paulo, SP, BR; II Curso de Medicina Veterinaria, Universidade de Santo Amaro, Sao Paulo, SP, BR; III Laboratorio Nacional de Biociencias – LNBio, Campinas, SP, BR

**Keywords:** Hyperadrenocorticism, *CRHR1*, Mutation, Cushing’s Disease, Dogs

## Abstract

**OBJECTIVES:**

Pituitary-dependent hyperadrenocorticism is the most common cause of naturally occurring hypercortisolism in dogs. *CRHR1* expression in human and dog corticotrophinomas suggested that this gene affects pituitary tumorigenesis. The present study aimed to investigate mutations in the *CRHR1* coding region in poodles with pituitary-dependent hyperadrenocorticism.

**METHODS:**

Fifty poodles with pituitary-dependent hyperadrenocorticism and 50 healthy poodles were studied. Genomic DNA was amplified by PCR and analyzed by Sanger sequencing.

**RESULTS:**

The novel *CRHR1* p.V97M mutation was identified in one dog. This valine residue, located in the amino-terminal extracellular domain, exhibits high affinity for its corticotropin-releasing hormone (CRH) ligand. Bioinformatic analysis revealed structural rearrangements in the mutant protein, with a 17% increase in the surface binding affinity between CRHR1 and CRH. *In vitro* functional studies showed that mutant CRHR1 induced higher ACTH secretion than the wild type after stimulation with human CRH.

**CONCLUSION:**

These results suggest that germline activating mutations in *CRHR1* may be a rare cause of pituitary hyperadrenocorticism in poodles.

## INTRODUCTION

Cushing’s disease is a common endocrinopathy in dogs and is characterized by polydipsia, polyuria, polyphagia, abdominal enlargement and alopecia 1-3. Pituitary-dependent hyperadrenocorticism (PDH) is the most common cause of naturally occurring hypercortisolism in dogs, accounting for 80 to 85% of all cases. PDH mainly affects dogs aged 6 years and older, and poodles, dachshunds, shih tzus and various terrier breeds appear to be at the greatest risk 4, 5. More than 90% of dogs with PDH have pituitary tumors, most of which are microadenomas (<10 mm in diameter) 1, 3.

The secretion of ACTH is mainly controlled by corticotropin-releasing hormone (CRH), which binds to its receptor CRHR1 and activates the transcription of proopiomelanocortin (POMC) to release ACTH. The expression of these factors is regulated by glucocorticoids. Other hypothalamic compounds, such as vasopressin, can also stimulate ACTH release but with much lower potency 6.

The finding that CRHR1 is expressed in corticotroph adenoma cells suggests that this receptor might play a role in pituitary tumorigenesis. Human patients with Cushing’s disease exhibit ACTH hypersecretion in response to a CRH stimulation test, despite the autonomous secretion of ACTH 7, 8. Similarly, *in vitro* studies have shown that adenomatous corticotroph cells exhibit a dose-dependent increase in ACTH levels after CRH stimulation 9, 10. Additionally, rat models treated with high doses of CRH developed corticotroph hyperplasia 11, 12. Therefore, multiple lines of evidence indicate that chronic and excessive hypothalamic hormone stimulation can result in increased pituitary hormone secretion and cell proliferation, leading to tumor development and neoplastic transformation in the adenohypophysis 13.

In dogs with PDH, the findings have been somewhat conflicting, with different studies reporting hyposecretion, normal secretion or hypersecretion of ACTH in response to CRH. However, dogs with PDH generally exhibit a persistent response to CRH, with elevated ACTH 14-16.

Considering the above evidence of the role of CRH and its receptor CRHR1 in pituitary tumor development and the high incidence of PDH in poodle dogs, our aim was to screen for *CRHR1* germline mutations in poodles with PDH.

## MATERIALS AND METHODS

### Animals

This prospective observational study included dogs that were initially evaluated at the Veterinary Medical Teaching Hospital of the University of Guarulhos from April 2006 through September 2007. All dogs were enrolled in the study with the informed consent of their owners. The possibility of PDH was based on historical data and a physical examination.

The inclusion criteria were that each dog must have had at least two clinical signs identified by the owner (polyuria, polydipsia, polyphagia or abdominal enlargement) and at least 2 of the following 6 clinicopathological findings: high serum alkaline phosphatase activity, high serum alanine aminotransferase activity, hypercholesterolemia, hypertriglyceridemia, urine specific gravity <1.020, and thrombocytosis 1, 4. All dogs underwent abdominal ultrasonography. For each dog, the results of a low-dose dexamethasone suppression test (LDDST) were consistent with hyperadrenocorticism (serum cortisol concentrations 8 hours after dexamethasone treatment ≥1.4 μg/dL). The diagnostic criteria for PDH included the following: an LDDST result indicative of spontaneous hyperadrenocorticism, ultrasonographic evidence of bilateral adrenal enlargement showing 2 relatively equal-sized, homogeneous adrenal glands and the absence of an adrenal mass, and plasma concentration of endogenous ACTH >17 pg/mL 17, 18.

Computed tomography (CT) and magnetic resonance imaging (MRI) were not performed due to the cost of these examination, the need for anesthesia and because none of the dogs had signs of neurological conditions or suspicion of pituitary macroadenoma.

All dogs with concurrent disorders were excluded from the study, as were those with an incomplete diagnosis of PDH, those with adrenal-dependent hyperadrenocorticism suspicion, and those that recently underwent PDH treatment.

### Endocrine tests and hormone assays

For the LDDST, blood samples (2 mL each) were collected before and 8 hours after IV administration of 0.01 mg/kg dexamethasone to determine the serum concentrations of cortisol. A serum cortisol concentration ≥1.4 μg/dL at 8 hours after dexamethasone administration was considered consistent with naturally occurring hyperadrenocorticism 2. Serum cortisol measurements were performed at the PROVET Hormone Laboratory in São Paulo, Brazil. A radioimmunoassay was performed using a solid-phase commercial diagnostic kit for cortisol (Coat-A-Count, Siemens Medical Solutions Diagnostics, Malvern, PA, USA) that was previously validated for canine cortisol 19. The intra-assay and inter-assay coefficients of variation for cortisol were <5.1% and <6.4%, respectively.

Plasma samples for ACTH measurements were collected in plastic tubes containing EDTA and immediately refrigerated. The samples were transported to the laboratory inside a Styrofoam container with ice for a maximum period of 1h before subsequent centrifugation at 1600 g for 15 min at 4°C. The samples were stored at -70°C until the assay was performed. Plasma samples were collected over 18 months and measured using 10 assays. Plasma ACTH concentrations were measured using an immunoradiometric assay (IRMA) with a commercial kit (ELSA-ACTH, CisBio International, Codolet, France). The ACTH intra-assay and inter-assay coefficients of variation according to the manufacturer were <6.1% and <5.3%, respectively; the intra-assay and inter-assay coefficients obtained in the laboratory were <7.8% and <9.9%, respectively. Plasma ACTH measurements were performed at the Hormone and Molecular Genetics Laboratory of Clinics Hospital, School Medicine, University of São Paulo, Brazil. We used a human ACTH kit due to the lack of a specific kit for dogs and because the ACTH molecule is highly homologous between these two species.

The control group comprised 50 healthy poodles, including 32 females and 18 males with a mean age of 9.4±2.8 years (range, 6 to 16 years), which presented to the Veterinary Teaching Hospital for ophthalmopathies, skin lesions or elective castration. Control dogs were matched to PDH dogs according to age (≥6 years) and breed. The criteria for inclusion in the control group were that each dog was healthy, not obese, and presenting with no clinical signs suggestive of hyperadrenocorticism and with normal routine biochemical tests (urea, creatinine, alanine aminotransferase, alkaline phosphatase) and hemogram.

#### *CRHR1* sequencing and analysis

Genomic DNA was extracted from peripheral blood leukocytes that were obtained from the 50 PDH and 50 control poodles using standardized protocols 20. The *CRHR1* coding region and intron-exon junctions (GenBank NC_006591.3) were amplified by PCR in an automated thermal cycler (Applied Biosystems PCR System 9700) using specific primers. The PCRs were performed with 2 µL of genomic DNA in a 50-µL final volume that contained 200 µM dNTPs, 0.5 mM (20 pmol) primers, 1.5 U of GoTaq Polymerase (Promega Corporation, Madison, WI, USA), 10 µL of 5x PCR reaction buffer (Promega), 1.5 mM MgCl_2_ and 0.4 M betaine. The PCR programs included an initial activation at 98°C for 5 min followed by 40 cycles of 45 s at 98°C, 30 s at 50-58°C (depending on the primers) and 45 s at 72°C, with a final extension at 72°C for 5 min. The final extension step for larger fragments, namely exons 6-8 (993 bp) and 9-12 (1,235 bp), was 72°C for 3 min. The products were visualized on a 1.5% agarose gel containing ethidium bromide.

Eight pairs of intron-flanking primers were designed based on the intron/exon structure of the *CRHR1* gene using the Primer3 core program. Exons 6 to 8 and exons 9 to 12 were amplified as single fragments (Table 1).

The resulting amplified products were purified and subjected to capillary electrophoresis in an automatic ABI3100 Genetic Analyzer (Applied Biosystems). The resulting sequences were compared with the *CRHR1* reference sequence and published on the NCBI website under Ensembl accession number ENSCAFT00000021552.

#### *CRHR1* allelic variant analysis by restriction enzyme digestion

The allelic variant in exon 4 (codon 97) of the *CRHR1* gene eliminates a BstUI cleavage site. To identify this variant in the control group, we performed enzymatic digestion. Digestion reactions were performed on all samples in the control group.

#### Molecular modeling of *CRHR1*

A homology model for the mutant canine CRHR1 was constructed based on an available crystal structure (PDB ID: 3EHT), which includes the human extracellular domain of CRHR1 bound to the CRH peptide template 21, using YASARA 22. The p.V97M mutant complex was constructed by superimposing the modeled structure of the mutant canine CRHR1 over the structure of the wild-type human CRHR1-CRH complex. To assess whether the mutation interferes with ligand-receptor binding, structure optimization was performed by energy minimization to estimate the binding energy between the hormone and the receptor. The binding energy was calculated using YASARA (See: http://www.yasara.org) with the YAMBER3 force field 22.

#### *In vitro* studies

Plasmid construction: The human CRHR1 cDNA sequence in the pcDNA plasmid was purchased from LabLife (Cambridge, MA, USA). The plasmid was mutagenized using specific primers (sense, 5′-CTGGGCCGCCCGCATGAATTACTCCG-3′ and anti-sense, 5′-CGGAGTAATTCATGCGGGCGGCCCAG-3′) with the QuikChange Mutagenesis Kit (Stratagene, La Jolla, CA, United States), according to the manufacturer’s instructions. The presence of the mutagenic insertion was confirmed by automated sequencing using a pcDNA primer (5′-TAGAAGGCACAGTCGAGG-3′).

Cell culture: AtT20 cells were grown in DMEM (Invitrogen) supplemented with 10% fetal bovine serum (Invitrogen) and 1x antibiotics (Invitrogen) at 37°C with 5% CO_2._ The AtT20 cells were seeded at a density of 5x10^4^ cells per well in 24-well plates 1 day before transfection. The cells were transfected with 1,000 ng/well of plasmid DNA. The empty pcDNA vector was used to reach the total amount of plasmid per well.

Transient transfection: In each well, 1 µg of DNA was diluted in 150 mM NaCl to a final volume of 50 µL, and 2 µL of jetPEI reagent was diluted separately in 150 mM NaCl to a final volume of 50 µL. These 2 reagents were mixed and incubated for 20 min at room temperature. The jetPEI/DNA mixture (100 µL) was added to AtT20 cells that were seeded in 500 µL of serum-containing medium; the transfection reagent and medium were homogenized by gently swirling the plate. The transfected cells were incubated in a cell culture incubator for 24h. The cells were then washed 3 times with PBS, and 100 nM human CRH (hCRH; Ferring) was added to each well. Aliquots (10 µL) of the supernatant media were collected from transfected AtT20 cells for ACTH measurement 6, 12 and 24h after hCRH addition. The assays were performed twice in triplicate. The ACTH levels in media from transiently transfected mutant and wild-type cells were expressed in terms of the fold change over the basal ACTH level in non-stimulated cells that were transfected with empty vector.

### Statistical analysis

Analysis of variance (ANOVA) followed by Bonferroni’s adjustment was used to compare the normalized fold change in ACTH among groups at each time point. The data were analyzed using Sigma Stat 3.5 software. Statistical significance was set at *p*<0.05.

## RESULTS

### Demographic and general characteristics of the animals

The study included 50 consecutively selected poodles with PDH, including 33 females and 17 males with a mean age of 8.7±2.8 years (range, 1.5 to 14 years), which presented at the Veterinary Hospital of Guarulhos University. Among the 50 dogs, 3 had family members that were also affected by PDH. The initial laboratory screening tests consisted of determining the serum levels of cholesterol, triglycerides, alkaline phosphatase, and alanine aminotransferase as well as tests for glycemia, urinary density, dexamethasone suppression, and abdominal ultrasonography.

Hypercholesterolemia and hypertriglyceridemia were found in 78% (n=39/50) and 74% (n=37/50) of the cases, respectively, with mean cholesterol values of 396 ± 156 mg/dL and triglycerides of 169±96 mg/dL. Elevated blood levels of alkaline phosphatase and alanine aminotransferase were observed in 78% (n=39/50) of the cases (mean value 459±401 IU/L) and in 70% (n=35/50) of the cases (mean value 204±158 IU/L), respectively. Urinary densities below the reference values (1.025–1.045) were identified in 80% of the animals (n=40/50), of which 20% (n=10/50) showed hyposthenuria (density≤1008). Hyperglycemia was identified in six animals (12%), two of which were insulin-dependent diabetics.

No animals showed suppression of serum cortisol levels 8 hours after the intravenous application of dexamethasone (mean values of cortisol=5.4±3.3 μg/dL), confirming endogenous hypercortisolism. All cases were diagnosed with PDH based on ACTH baseline plasma concentrations above 17 pg/mL (mean values of ACTH=42±30 pg/mL).

Abdominal ultrasound showed symmetric adrenal glands with regular contours, homogeneous echotextures, and the absence of nodulations and calcifications. The mean values of the caudal pole thickness of the right and left adrenal glands were 0.73±0.14 and 0.74±0.11 cm, respectively. Dogs with pituitary-dependent hypercortisolism have, on average, a caudal pole thickness greater than or equal to 0.7 cm 23.

### Mutation analysis

**Figure 1:**
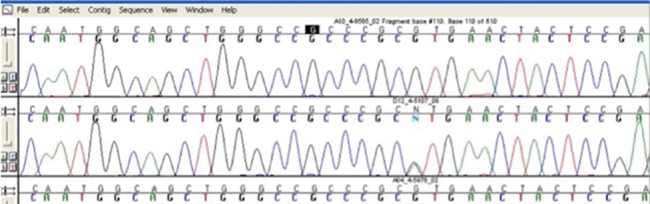
The wild-type sequence of exon 4 of the *CRHR1* gene from a control dog (upper panel) and the heterozygous G-to-A substitution causing the amino acid change (p.V97M) in a poodle with ACTH-dependent hypercortisolism (lower panel).

**Figure 2:**
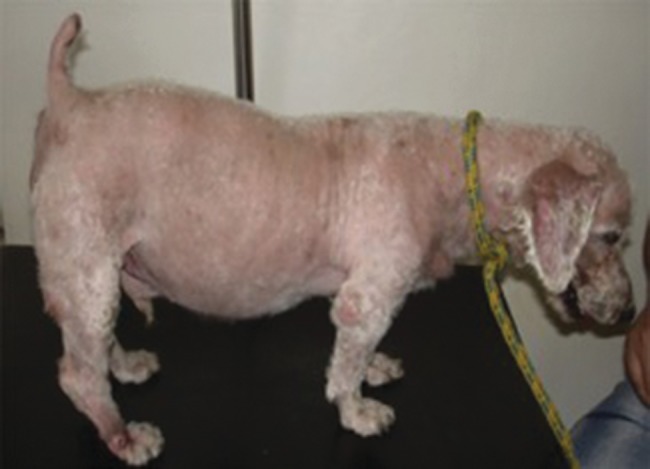
The dog with ACTH-dependent hypercortisolism and carrying the allelic variant p.V97M, showing marked abdominal distension, alopecia, skin atrophy and telangiectasia.

We identified only one heterozygous variant in one of the dogs with PDH, in exon 4 of the *CRHR1* gene. This variant results from a guanine-to-adenine (G → A) change in codon 97 that causes a valine-to-methionine substitution (p.V97M) (Figure 1). Although both valine (encoded by GTG) and methionine (encoded by ATG) are nonpolar amino acids, the Val97 residue is highly conserved among different species. According to BLAST (https://blast.ncbi.nlm.nih.gov/Blast.cgi), the coding region of *CRHR1* is 98% similar between dogs and humans, and the protein shares the same amino acid, valine, at position 97. Moreover, this change occurred in the amino-terminal portion of the extracellular domain of the CRHR1 receptor, which has a high affinity for the ligand 22. The p.V97M allelic variant was not found among the 100 poodle alleles in the control group or in the normal siblings of the disease cases. The animal with the p.V97M allelic variant was 9 years old and had a history suggestive of hypercortisolism for at least 12 months. The dog showed clinical features of hyperadrenocorticism, suggesting chronic and progressive disease (Figure 2). The serum cortisol level after an intravenous injection of dexamethasone was 7.8 μg/dL, and his baseline plasma ACTH level (45 pg/mL) confirmed the presence of ACTH-dependent hypercortisolism. An abdominal ultrasound examination revealed thickening of the caudal poles of the right (1.0 cm) and left (0.92 cm) adrenal glands, both of which showed regular contours, homogeneous echotexture and reduced echogenicity.

The animal was treated with mitotane at a dose of 25 mg/kg every 12h for 7 days (induction phase); subsequently, it was treated with 25 mg/kg twice a week (maintenance phase). At this time, trilostane, which is currently considered the first choice of drug in canine PDH therapy, was not available in Brazil. The animal showed partial improvement, with several adjustments of the mitotane dose being necessary during the 10-months of follow-up therapy. For personal and financial reasons, the owner discontinued the treatment. The animal died 6 months after the treatment was discontinued, likely due to pulmonary embolism.

The affected dog’s male offspring is not a carrier of the p.V97M mutation and did not shown symptoms of Cushing’s disease at 7 years of age.

### Mutant protein modeling study

The allelic variant p.V97M is located in the extracellular amino terminus of the CRHR1 protein, which is considered the primary region for receptor-ligand binding 21, 24. Protein modeling revealed that relative to the wild-type protein, the CRHR1 mutant showed a 17% increase in its binding affinity for CRH. Changes in the 3-dimensional structure of the extracellular domain of CRHR1 affect receptor activation and affinity for the ligand 21, 25.

**Figure 3:**
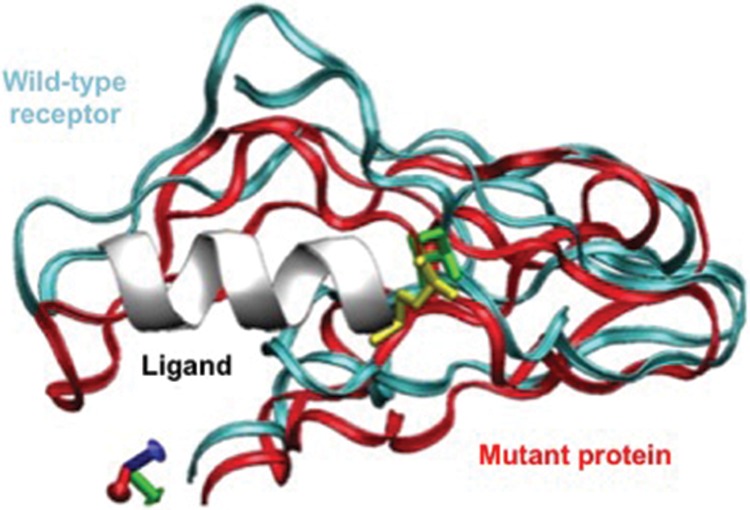
The structural rearrangement in the mutant protein (red) and the resulting changes in the contact surface between the ligand and the mutant receptor (containing methionine). The wild-type receptor is shown for comparison.

The quaternary structures of the mutant and wild-type proteins were examined, and the binding energy between the hormone and its receptors was evaluated to better understand the involvement of this variant in the pathogenesis of ACTH-dependent hyperadrenocorticism. A comparison of the 3-dimensional structures of the complexes formed with the mutant (p.V97M) and wild-type proteins revealed a structural rearrangement in the contact surface between the p.V97M receptor and the ligand (Figure 3).

To assess whether the mutation interferes with receptor-ligand binding, optimization of the mutant complex structure was performed using energy minimization. The binding energy between the hormone and the receptor was then estimated.

The binding enthalpy was 1,893 kJ/mol for the wild-type complex and 2,134 kJ/mol for the p.V97M complex; the mutant CRHR1 exhibited a 17% increase in its binding affinity for CRH, therefore suggesting a clear gain of function for the p.V97M variant.

### Functional characterization of the CRHR1 mutant protein

**Figure 4:**
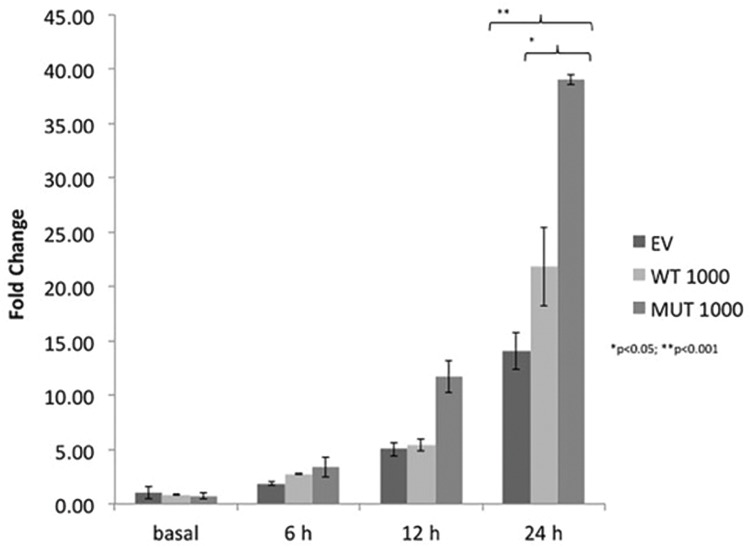
The observed fold changes in ACTH relative to the basal ACTH level in cells transfected with empty vector were 5.01±0.65 (empty vector, EV), 5.41±0.57 (wild-type, WT) and 11.57±1.45 (mutant, MUT) at 12h and 14.0±1.72 (EV), 21±3.6 (WT) and 38.9±0.45 (MUT) at 24h. There were significant differences in the ACTH levels of cells transfected with the mutant plasmid compared with cells transfected with the wild-type plasmid or with empty vector at 24h (**p<0.01, *p<0.05).

The fold changes in the ACTH levels after stimulation relative to the basal levels in AtT20 cells transfected with empty, wild type and mutant vectors were 5.01±0.65, 5.41±0.57 and 11.57±1.45, respectively, at 12 h and 14.0±1.72, 21±3.6 and 38.9±0.45, respectively, at 24h. There was a significant increase in the ACTH levels in the mutant relative to the wild-type at 12 h (*p<0.05) and 24 h (**p<0.01) (Figure 4).

## DISCUSSION

The expression of CRHR1 in human corticotrophinomas suggests that hormone receptors play a role in pituitary tumorigenesis, promoting sustained cell stimulation even in the absence of hypothalamic hormones 26-28. Corticotroph tumors in dogs and humans exhibit CRHR1 overexpression and greater sensitivity to CRH 13, 29, 30.

In the present study, we screened the *CRHR1* gene for mutations in poodles with PDH and identified 1 dog with a novel p.V97M activating heterozygous allelic mutation that was located at the amino-terminal portion of the extracellular domain of CRHR1. The p.V97M CRHR1 mutant has a larger apolar surface created by the methionine side chain, which may increase the van der Waals contacts with the Ile41 residue of CRH and thus favor ligand binding. Optimization of the molecular and quaternary structure of this mutated protein by energy minimization indicated the existence of a structural rearrangement due to an altered contact surface between the mutated receptor and the ligand. *In vitro* studies revealed that AtT20 cells transfected with the mutant plasmid over-secreted ACTH compared relative to cells transfected with the wild-type plasmid. The maximal effects of the mutant and wild-type plasmids on hormone release were observed after 24 h of hCRH stimulation, which caused approximately 38.9- and 21-fold increases in ACTH levels, respectively, over the basal levels in cells transfected with empty vectors. This result confirmed that the variant is an activating mutation.

Changes in the 3-dimensional structure of the extracellular domain of CRHR1 affect the activation of the receptor and its affinity for the ligand 21, 24, 31, 32.

The binding of CRH to CRHR1 ligand-binding sites has been extensively investigated in studies of chimeric receptors. These studies have indicated that residues 1-118 of the N-terminal domain (specifically, amino acid residues 43-50 and 76-84 in humans and 68-109 in mice) are the most important regions for agonist binding 33-36.

Pioszak, Parker 21 reported the importance of valine 97 (Val97) for interactions between CRHR1 and CRH. This interaction occurs through 2 intermolecular hydrogen bonds between the oxygen and nitrogen atoms of the amide C-terminal ligand (CHR) and the amidic nitrogen atoms and carbonyl oxygen of the main chain of the receptor (residue Val97).

Optimization of the molecular and quaternary structure of the mutant protein by energy minimization indicated the occurrence of a structural rearrangement due to an altered contact surface between the mutant receptor and the ligand. The p.V97M *CRHR1* mutant has a larger apolar surface that is created by the methionine side chain, which may increase van der Waals contacts with the Ile41 residue of CRH to favor binding. Additionally, the prediction that the surface binding energy is increased between the ligand (CRH) and the mutant receptor (CRHR1m) supports the hypothesis that this activating mutation in CRHR1 is involved in the pathogenesis of PDH in this poodle. The late onset of PDH in this animal may contradict the notion that the disease has a genetic etiology; however, other model germline disorders that present later in life, including retinitis pigmentosa and multiple endocrine neoplasia, are well accepted 37, 38. In the affected poodle, the increased ACTH stimulation and secretion caused by the increased affinity of the mutant CRHR1 for CRH might have taken several years to promote corticotroph hyperplasia. Unfortunately, this possibility remains untested, as neither skull magnetic resonance (to visualize the pituitary mass) nor animal necropsy (to identify corticotroph hyperplasia or neoplasia) was performed.

Thyrotropin receptors (TSHRs) have been widely studied as models for activating mutations, which are located not only in the transmembrane region but also in the amino-terminal extracellular domain 39. Cases of non-autoimmune congenital hyperthyroidism have been reported as secondary to *de novo* germline mutations in the TSHR extracellular domain in humans 40. In addition to the influence of the extracellular amino-terminal domain of GPCRs on receptor-ligand binding affinity, this domain might also act as an internal antagonist that is capable of having agonist or antagonist properties depending on whether it is bound to the ligand 39.

Although the monoclonal origin of pituitary adenomas has been well established since 1990, there have been instances in which the monoclonal character was acquired at a later stage of polyclonal growth 41. Schulte, Oldfield 42 demonstrated that corticotroph adenomas of the pituitary gland might arise from a single cell or from more than one cell, making them monoclonal or polyclonal in origin. These observations are compatible with the idea of an initiating stimulus that causes hyperplasia of specific cell types in the pituitary, itself giving rise to several distinct clones with variable potential to develop into tumors. Such stimuli may include hypothalamic trophic factors, intrapituitary growth factors or pituitary-specific oncogenes 43.

Another hypothesis is that the affected dog, by carrying a *CRHR1* activating mutation, developed only pituitary hyperplasia and not an adenoma. Pituitary hyperplasia and pituitary adenoma are both possible explanations for the ACTH over-secretion and chronic hypercortisolism observed in this dog.

This study aimed to investigate whether Cushing’s disease in poodles has a genetic origin. We found only one dog with an activating mutation among 50 poodles with hyperadrenocorticism, which indicated that in most of cases, the mutation did not cause Cushing’s disease. However, taking into account the high similarity of CRHR1 between humans and dogs, our data suggest that this mutation may also be involved in some human cases of Cushing’s disease; thus, these results may be clinically relevant.

The limitations of this study included the unavailability of tumor tissues and the availability of only one male offspring from the dog with the allelic variant p.V97M to test for segregation.

In conclusion, we reported a mutation in *CRHR1* that leads to persistent activation of the receptor in a poodle with PDH, indicating that this is a rare cause of ACTH-dependent hypercortisolism. This new finding, although rare, suggests that other germline mutations that activate the hypothalamuspituitary-adrenal axis may be associated with this phenotype in dog breeds commonly affected by ACTH-dependent hyperadrenocorticism.

## AUTHOR CONTRIBUTIONS

De Marco V performed clinical evaluations and genetic studies. Guzzo MF and Gomes LG performed *in vitro* studies. Oliveira PS performed bioinformatic analysis. Gomes LG, Carvalho LR and Mendonca BB supervised the work.

## Figures and Tables

**Table 1:** Forward (F) and reverse (R) primers for canine *CRHR1* amplification, with product sizes (bp) and annealing temperatures (T_a_).

Primer	Sequence	Product size (bp)	T_a_ (°C)
1F	5′ CTGAGTCAGGAGACGGCGCA 3′	422	50
1R	5′ TCATTGTGGCGAAGCTGCTG 3′		
2F	5′ TTGGGATCCTAGGCTTGATG 3′	413	58
2R	5′ GCAAGATTCCAGGTCTCCAG 3′		
3F	5′ GAACGAATGCTGGATCCCTA 3′	456	50
3R	5′ TCTCGTGGATACAACCAC 3′		
4F	5′ AGATGGACGAACCAATGGAC 3′	581	58
4R	5′ AGTCTCCTTCCCACCCTGTC 3′		
5F	5′ GGGAAGGGGAATAACTACCG 3′	401	50
5R	5′ GTCTCTTGGAACCTCTGGCA 3′		
6-8F	5′ TCCTCAGTTTCCCCATCCATA 3′	993	50
6-8R	5′ TGAGGTACAGGCTCTCAGCC 3′		
9-12F	5′ GAGGCATTGTGTTGGGATCT 3′	1,235	55
9-12R	5′ CAGGTCCCTAATGAGGATGC 3′		
13F	5′ ACGTGTACTGCTGCTTGTGG 3′	419	58
13R	5′ GAGAGCAGCCATAGTCTGGG 3′		

## References

[1] Feldman EC, Nelson RW, Reusch CE, Scott-Moncrieff JCR, Behrend EN (2015). Canine and feline endocrinology and reproduction.

[2] Behrend EN, Kooistra HS, Nelson R, Reusch CE, Scott-Moncrieff JC (2013). Diagnosis of spontaneous canine hyperadrenocorticism: 2012 ACVIM consensus statement (small animal). J Vet Intern Med.

[3] Peterson ME (2007). Diagnosis of hyperadrenocorticism in dogs. Clin Tech Small Anim Pract.

[4] Kooistra HS, Galac S (2010). Recent advances in the diagnosis of Cushing’s syndrome in dogs. Vet Clin North Am Small Anim Pract.

[5] Ling GV, Stabenfeldt GH, Comer KM, Gribble DH, Schechter RD (1979). Canine hyperadrenocorticism: pretreatment clinical and laboratory evaluation of 117 cases. J Am Vet Med Assoc.

[6] Seasholtz A (2000). Regulation of adrenocorticotropic hormone secretion: lessons from mice deficient in corticotropin-releasing hormone. J Clin Invest.

[7] Hermus AR, Pieters GF, Pesman GJ, Smals AG, Benraad TJ, Kloppenborg PW (1986). Responsivity of adrenocorticotropin to corticotropin-releasing hormone and lack of suppressibility by dexamethasone are related phenomena in Cushing’s disease. J Clin Endocrinol Metab.

[8] Takeda R, Ito T, Kawato M, Nakabayashi H, Miyamori I, Morise T (1986). ACTH secretory responsiveness of pituitary adrenotroph cell tumor to adrenocorticotropin-releasing factor in Cushing’s disease and Nelson’s syndrome. Exp Clin Endocrinol.

[9] Grino M, Boudouresque F, Conte-Devolx B, Gunz G, Grisoli F, Oliver C (1988). In vitro corticotropin-releasing hormone (CRH) stimulation of adrenocorticotropin release from corticotroph adenoma cells: effect of prolonged exposure to CRH and its interaction with cortisol. J Clin Endocrinol Metab.

[10] Horvath SE, Asa SL, Kovacs K, Adams LA, Singer W, Smyth HS (1990). Human pituitary corticotroph adenomas in vitro: morphologic and functional responses to corticotropin-releasing hormone and cortisol. Neuroendocrinology.

[11] Gertz BJ, Contreras LN, McComb DJ, Kovacs K, Tyrrell JB, Dallman MF (1987). Chronic administration of corticotropin-releasing factor increases pituitary corticotroph number. Endocrinology.

[12] McNicol AM, Kubba MA, McTeague E (1988). The mitogenic effects of corticotrophin-releasing factor on the anterior pituitary gland of the rat. J Endocrinol.

[13] Asa SL (1991). The role of hypothalamic hormones in the pathogenesis of pituitary adenomas. Pathol Res Pract.

[14] Orth DN, Peterson ME, Drucker WD (1988). Plasma immunoreactive proopiomelanocortin peptides and cortisol in normal dogs and dogs with Cushing’s syndrome: diurnal rhythm and responses to various stimuli. Endocrinology.

[15] Peterson ME, Kemppainen RJ, Orth DN (1992). Effects of synthetic ovine corticotropin-releasing hormone on plasma concentrations of immunoreactive adrenocorticotropin, alpha-melanocyte-stimulating hormone, and cortisol in dogs with naturally acquired adrenocortical insufficiency. Am J Vet Res.

[16] van Wijk PA, Rijnberk A, Croughs RJ, Wolfswinkel J, Selman PJ, Mol JA (1994). Responsiveness to corticotropin-releasing hormone and vasopressin in canine Cushing’s syndrome. Eur J Endocrinol.

[17] Feldman EC (1983). Distinguishing dogs with functioning adrenocortical tumors from dogs with pituitary-dependent hyperadrenocorticism. J Am Vet Med Assoc.

[18] Gould SM, Baines EA, Mannion PA, Evans H, Herrtage ME (2001). Use of endogenous ACTH concentration and adrenal ultrasonography to distinguish the cause of canine hyperadrenocorticism. J Small Anim Pract.

[19] Watson AD, Church DB, Emslie DR (1993). Plasma cortisol concentrations in dogs given cortisone or placebo by mouth. Res Vet Sci.

[20] Miller SA, Dykes DD, Polesky HF (1988). A simple salting out procedure for extracting DNA from human nucleated cells. Nucleic Acids Res.

[21] Pioszak AA, Parker NR, Suino-Powell K, Xu HE (2008). Molecular recognition of corticotropin-releasing factor by its G-protein-coupled receptor CRFR1. J Biol Chem.

[22] Krieger E, Darden T, Nabuurs SB, Finkelstein A, Vriend G (2004). Making optimal use of empirical energy functions: force-field parameterization in crystal space. Proteins.

[23] Barthez PY, Nyland TG, Feldman EC (1995). Ultrasonographic evaluation of the adrenal glands in dogs. J Am Vet Med Assoc.

[24] Perrin MH, Grace CR, Digruccio MR, Fischer WH, Maji SK, Cantle JP (2007). Distinct structural and functional roles of conserved residues in the first extracellular domain of receptors for corticotropin-releasing factor and related G-protein-coupled receptors. J Biol Chem.

[25] Teli T, Markovic D, Hewitt ME, Levine MA, Hillhouse EW, Grammatopoulos DK (2008). Structural domains determining signalling characteristics of the CRH-receptor type 1 variant R1beta and response to PKC phosphorylation. Cell Signal.

[26] Chen R, Lewis KA, Perrin MH, Vale WW (1993). Expression cloning of a human corticotropin-releasing-factor receptor. Proc Natl Acad Sci U S A.

[27] Suda T, Tozawa F, Dobashi I, Horiba N, Ohmori N, Yamakado M (1993). Corticotropin-releasing hormone, proopiomelanocortin, and glucocorticoid receptor gene expression in adrenocorticotropin-producing tumors in vitro. J Clin Invest.

[28] Sakai Y, Horiba N, Sakai K, Tozawa F, Kuwayama A, Demura H (1997). Corticotropin-releasing factor up-regulates its own receptor gene expression in corticotropic adenoma cells in vitro. J Clin Endocrinol Metab.

[29] Dieterich KD, Gundelfinger ED, Lüdecke DK, Lehnert H (1998). Mutation and expression analysis of corticotropin-releasing factor 1 receptor in adrenocorticotropin-secreting pituitary adenomas. J Clin Endocrinol Metab.

[30] Teshima T, Hara Y, Takekoshi S, Teramoto A, Osamura RY, Tagawa M (2009). Expression of genes related to corticotropin production and glucocorticoid feedback in corticotroph adenomas of dogs with Cushing’s disease. Domest Anim Endocrinol.

[31] Dautzenberg FM, Kilpatrick GJ, Wille S, Hauger RL (1999). The ligand-selective domains of corticotropin-releasing factor type 1 and type 2 receptor reside in different extracellular domains: generation of chimeric receptors with a novel ligand-selective profile. J Neurochem.

[32] Perrin MH, Grace CR, Riek R, Vale WW (2006). The three-dimensional structure of the N-terminal domain of corticotropin-releasing factor receptors: sushi domains and the B1 family of G protein-coupled receptors. Ann N Y Acad Sci.

[33] Liaw CW, Grigoriadis DE, Lovenberg TW, De Souza EB, Maki RA (1997). Localization of ligand-binding domains of human corticotropin-releasing factor receptor: a chimeric receptor approach. Mol Endocrinol.

[34] Wille S, Sydow S, Palchaudhuri MR, Spiess J, Dautzenberg FM (1999). Identification of amino acids in the N-terminal domain of corticotropin-releasing factor receptor 1 that are important determinants of high-affinity ligand binding. J Neurochem.

[35] Hillhouse EW, Grammatopoulos DK (2006). The molecular mechanisms underlying the regulation of the biological activity of corticotropin-releasing hormone receptors: implications for physiology and pathophysiology. Endocr Rev.

[36] Grace CR, Perrin MH, Gulyas J, Digruccio MR, Cantle JP, Rivier JE (2007). Structure of the N-terminal domain of a type B1 G protein-coupled receptor in complex with a peptide ligand. Proc Natl Acad Sci U S A.

[37] Wu DM, Khanna H, Atmaca-Sonmez P, Sieving PA, Branham K, Othman M (2010). Long-term follow-up of a family with dominant X-linked retinitis pigmentosa. Eye (Lond).

[38] Hoff AO, Hauache OM (2005). [Multiple endocrine neoplasia type 1 (MEN 1): clinical, biochemical and molecular diagnosis and treatment of the associated disturbances]. Arq Bras Endocrinol Metabol.

[39] Duprez L, Parma J, Costagliola S, Hermans J, Van Sande J, Dumont JE (1997). Constitutive activation of the TSH receptor by spontaneous mutations affecting the N-terminal extracellular domain. FEBS Lett.

[40] Gruters A, Schoneberg T, Biebermann H, Krude H, Krohn HP, Dralle H (1998). Severe congenital hyperthyroidism caused by a germ-line neo mutation in the extracellular portion of the thyrotropin receptor. J Clin Endocrinol Metab.

[41] Herman V, Fagin J, Gonsky R, Kovacs K, Melmed S (1990). Clonal origin of pituitary adenomas. J Clin Endocrinol Metab.

[42] Schulte HM, Oldfield EH, Allolio B, Katz DA, Berkman RA, Ali IU (1991). Clonal composition of pituitary adenomas in patients with Cushing’s disease: determination by X-chromosome inactivation analysis. J Clin Endocrinol Metab.

[43] Clayton RN, Farrell WE (2001). Clonality of pituitary tumours: more complicated than initially envisaged. Brain Pathol.

